# From Vision to Action: National Bleeding Disorders Foundation’s Roadmap for Achieving Health Equity, Diversity, and Inclusion

**DOI:** 10.1089/heq.2024.0146

**Published:** 2025-03-11

**Authors:** Keri L. Norris, Marissa Melton, Karina Lopez, Dawn Rotellini, Leonard A. Valentino

**Affiliations:** ^1^ National Bleeding Disorders Foundation, New York, New York, USA.; ^2^ Hemophilia and Thrombophilia Center, Rush University Medical Center, Chicago, Illinois, USA.

**Keywords:** health, equity, diversity, inclusion, bleeding disorders, community

## Abstract

**Background:**

The National Bleeding Disorders Foundation (NBDF) consistently hears from lived experience experts about daily challenges preventing them from leading their best life and thriving physically, mentally, and emotionally. Dedicated to enabling people and families impacted by inheritable blood and bleeding disorders (BDs) to thrive, NBDF recognized that the impact of social determinants, disparities, and inequities of health must be addressed explicitly to achieve their mission.

**Methods:**

NBDF developed a health equity, diversity, and inclusion strategic direction for the coming decade in the context of collaboration with regional, national, and international partners. Drawing upon limited available data, extensive community consultation, and a thorough landscape scan, NBDF identified specific social determinants of health preventing health equity in the inheritable BDs community.

**Results:**

NBDF developed a model detailing the engaging, empowering, and elevating work individual, community, organizational, and institutional stakeholders must undertake to dismantle health equity barriers. Overarching priorities and strategies were established, providing leadership, and support was offered in the form of tools, resources, and expertise.

**Conclusion:**

Designed to be tailored to needs and capacities, this approach may be applied by other rare disorder communities to develop and operationalize their own health, equity, diversity, and inclusion strategic direction to advance social justice.

## Introduction

Lived experience experts (LEEs), the people who live every day with any chronic lifelong disease/disorder, such as an inheritable blood or bleeding disorder (BD), are uniquely positioned to illuminate challenges with access to care and quality treatment, issues in accessing the health care system, barriers to prompt and optimal diagnosis and care, and impacts of social determinants of health (SDoH), disparities, and inequities.^1^ The National Bleeding Disorders Foundation (NBDF) consistently hears from LEEs about the challenges they face daily, which prevent them from thriving physically, mentally, and emotionally. NBDF is dedicated to finding cures for inheritable BDs and to addressing and preventing the complications of these disorders through research, education, and advocacy, enabling people and families to thrive.^2^ This mission implicitly encompasses the entire diverse inheritable BD community; however, implicit inclusion fails in the face of disparities and inequities. NBDF, therefore, developed a health equity, diversity, and inclusion (HEDI) strategic direction for the coming decade. The strategic direction provides vision, priorities, and concrete actions that enable all stakeholders, at all levels, to identify and address factors contributing to health inequities and disparities. It thus aims to actively advance health equity for all persons with inheritable BDs.

### Health equity and SDoH

The World Health Organization (WHO) defines health equity as achieved when everyone can attain their full potential for health and well-being.^3^ This requires removal of obstacles to health such as poverty, discrimination, and their consequences including powerlessness and lack of access to good jobs with fair pay, quality education and housing, safe environments, and high-quality health care.^4^ SDoH, often grouped into six categories (Table 1),^5–7^ are the nonmedical factors that influence health outcomes: the conditions in which individuals are born, grow, work, live, and age.^9^ They include wider forces and systems that shape daily life such as economic policies and systems, development agendas, social norms, social policies, racism, sexism, climate change, and political systems.^6^ Nonmedical factors account for 30–55% of health outcomes, a greater contribution than that of health sector factors.^6^

**Table 1.** Examples of Six Categories of SDoH

Category	Examples^5–7,8^
Economic stability	
	Unemployment and job insecurity Employment and job characteristics
	Income Intergenerational wealth
	Expenses
	Debt
	Medical bills
	Support
Neighborhood and physical environment	
	Housing availability, quality, overcrowding
	Transportation
	Safety
	Parks, green spaces
	Playgrounds
	Walkability
	Zip code/geography (rural vs. urban) Air and water quality, exposure to toxins Impacts of climate change
Education	
	Literacy
	Language
	Early childhood education
	Vocational training
	Higher education Equitable learning environments
Food	
	Hunger Food insecurity
	Access (proximity and affordability) to healthy options (e.g., food swamps, food deserts)
Community and social context	
	Social integration
	Support systems
	Community engagement Civic participation
	Discrimination Policies addressing racial and social justice Legal and criminal justice systems Income inequality Exposure to violence Stress
Health care system	
	Health insurance coverage, health care costs
	Provider availability
	Provider linguistic and cultural humility
	Health literacy Quality of care Guideline-concordant and evidence-based practice Timeliness of access, appointment duration Primary and specialty care access Referral completion

SDoH, social determinant(s) of health.

### Health inequity in the United States

The United States has a long history of health inequity with medical discrimination of individuals and groups based on gender, income, education, race/ethnicity, and other characteristics. The principles underpinning racism and slavery have been borne out in practices. Examples of such practices include mass sterilization of racialized women without consent or anesthesia, medical atrocities and experimental exploitation of enslaved and freed persons (e.g., the Tuskegee syphilis experiments), exclusion of Black students from medical schools and professional organizations, and segregation of Black patients.^10–14^ The 2018 U.S. economic burden of racial and ethnic health inequities was calculated to be as high as $451 billion, and of education-related health inequities, $978 billion.^15^

Disparities in research prioritization perpetuate disparities in care and outcomes. Some of the diseases for which research is least funded by the U.S. National Institutes of Health relative to disease burden affect primarily women.^16^ Trivialization of women’s health complaints is a common manifestation of ongoing gender bias in health care.^16^ U.S. maternal mortality rates are higher than in other developed nations and 2.5–3.1 times higher for non-Hispanic Black women than non-Hispanic White women.^17^ This trend may reflect the many social and political structures and policies born out of racism, classism, and gender oppression.^18^ The disproportionate vulnerability of lower- and middle-income families in the underfunded U.S. public health infrastructure was strikingly demonstrated during the COVID pandemic as millions lost their health insurance coverage with their jobs.^19^ Despite spending far more of its gross domestic product on health care, a 2021 analysis of 11 high-income countries ranked the performance of the U.S. health system last on access to care, administrative efficiency, equity, health care outcomes, and overall.^20^ A key feature distinguishing top-performing countries from the U.S. is investment in primary care systems to make high-value services equitably available to all individuals in all communities.^20^

Like the general population, health care providers (HCPs) hold negative explicit and implicit biases against many groups including racial and ethnic minoritized populations, disabled populations, gender and sexual minorities, those who are overweight/obese, have limited English proficiency, live with mental illness, or have lower socioeconomic status.^21,22^ Permeating the health care system, these biases impact patient–clinician communication, clinical decision making, level of care, and institutionalized practices.^21,23^ Efforts to raise awareness of HCP bias, engage HCPs in egalitarian goals for care delivery, and improve HCP diversity are often hindered by discriminatory policies and practices pervasive in many working and learning environments, as structural racism and implicit bias mutually reinforce one another.^22^ Long-standing norms, practices, policies, and structures often perceived as ordinary may reflect deeply ingrained racism.^24^ Even absent conscious intent to discriminate, unconscious or implicit bias may perpetuate discriminatory effects through deeply rooted attitudes and behaviors.^4,25^

### Health equity for persons with inheritable BDs

Hemophilia A and B are the two best-known inheritable BDs. These rare disorders are characterized by disproportionate bleeding, often into joints and vital organs, due to low levels or decreased function of coagulation factors VIII and IX, respectively.^26^ Prophylactic infusion of concentrates of replacement clotting factor every few days, starting from a young age,^27^ and a LEE-centric multidisciplinary team approach to care (e.g., as practiced in hemophilia treatment centers [HTCs])^28^ vastly improve the joint health and life expectancy of persons with hemophilia in high-income countries.^29–31^ Advances in hemophilia care include improved availability of safe replacement factor therapeutics, widespread adoption of multidisciplinary care models, and recent innovations such as therapeutic antibodies and gene therapy.^28,32–34^ Recent progress prompted a call for a paradigm shift in hemophilia treatment goals: from minimizing damaging bleeding events/sequelae and delaying death to a “functional cure” and health equity—a life unimpeded by hemophilia.^35^ This vision is ambitious but imaginable for some with full access to the latest advances and spurs research^26^ and advocacy efforts. For many persons with inheritable BDs, health equity remains unattainable. These include, but are not limited to, those with deficiencies of other clotting factors^36^ or mucocutaneous BDs^37^; women, girls, and persons who have or had the potential to menstruate (WGPPM)^38^; other minoritized and marginalized populations^39^; and the very large proportion of persons with hemophilia who cannot access the current standard of care, let alone recent innovations.^40^

A recent systematic review found that SDoH are associated with inferior health outcomes for persons with inheritable BDs and may influence the progression of their disorders.^41^ Rural living contributed significantly to delayed diagnosis and decreased access to care. This analysis of 13 articles highlighted the need to reduce economic burden through sustainable population health strategies and treatment options. It also identified addressing the physical, psychosocial, and emotional needs of persons with inheritable BDs through multidisciplinary comprehensive team care as a target priority.^41^ A qualitative survey of Canadian HCP perspectives also demonstrated an association between rural living and delayed diagnosis and suggested that socioeconomic status and race may influence access to care.^42^

The U.S. Centers for Disease Control and Prevention and American Thrombosis and Hemostasis Network (ATHN) collaboratively collect data on persons with inheritable BDs including race, ethnicity, sex, residential zip code, insurance status, and HIV infection status,^43^ but little research into outcome disparities has been reported. A recent exploratory study of the U.S. HTC registry reported improvements in hemophilia death rates between 1999 and 2020 among men of all races/ethnicities; however, the average age at death for Black males was 10 years younger than for their White counterparts (2010–2020).^44^ Fedewa et al.^44^ remark that HIV remained a leading cause of death in Black (but not White) men with hemophilia in 2020, and that in the general population, Black persons with HIV are less likely to be treated with antiretroviral drugs and their HIV to be well controlled.^45^ Much more research, investigating the full diversity of inheritable BDs and of persons with inheritable BDs, is required to identify and understand the SDoH impacting health equity in this community.

Historically, the diversity of inheritable BD research participants has not reflected the diversity of the community. Many interventional clinical trials involving persons with hemophilia in the clinicaltrials.gov database did not report race/ethnicity data until 2017,^46^ when the U.S. Food and Drug Administration began requiring it.^47^ The improved reporting then revealed significant underrepresentation of Black and Hispanic trial participants, with observed-to-expected ratios approximately 75% lower than census data.^46^ Lack of diversity among research participants limits the generalizability of trial results and access to life-saving treatments for the full community, with those experiencing the greatest health challenges often benefiting the least from representation.^48^

Individuals who menstruate, ovulate, and experience pregnancy are disproportionately affected by inheritable BDs due to the associated bleeding challenges, which remain under-researched and often misdiagnosed.^49–51^ Research has historically focused on hemophilia in boys and men, with much less study devoted to the more common von Willebrand disease (VWD), inherited equally by the sexes, and sexism in the field has long contributed to inequities for WGPPM.^52,53^ Many WGPPM experiencing multiple hemostatic challenges are not referred to a hematologist,^54,55^ and some report feeling that their symptoms are dismissed by HCPs, causing a burden of strenuous self-advocacy, especially around urgent care and invasive procedures.^38,54,56–58^ An average delay of 16 years between symptom onset and diagnosis for females with VWD has been reported in the U.S.^59^ In Europe, despite experiencing their first bleeding event at a similar age, the diagnostic delay for females with autosomal inheritable BDs was, on average, 6 years longer than for males.^49^ Labeling females with X-linked inheritable BDs as “carriers” is often misunderstood to equate asymptomatic, thus impeding diagnosis, appropriate care, and research.^53,60^

The rarity of studies on health inequities in the inheritable BD community, the lack of diverse representation in clinical trials, the personal intelligence shared by LEEs, and the evidence that SDoH impact equity in other areas of health all underscore the urgent need for concerted HEDI initiatives. In keeping with its mission of enabling people and families to thrive,^2^ NBDF developed a HEDI strategic direction for the coming decade.

### Examples of health equity initiatives

A 2008 WHO Commission called on all governments to lead global action on SDoH with the aim of achieving health equity.^61^ Numerous governmental and other organizations have taken up this challenge (Table 2). Each of these organizations provides important resources and drives invaluable initiatives in the pursuit of health equity. NBDF exists in partnership with a network of over 50 local, community-based organizations known as Chapters across the United States, some of which are independently operated, providing a variety of education and services to the inheritable BD community.^79^ The NBDF HEDI strategic direction was developed in the context of collaboration with all these important partners.

**Table 2.** Examples of Health Equity Initiatives

Organization	Guiding principle(s)	Strategy/focus	Example(s)	Reference(s)
National Institute for Health and Care Excellence (NICE, United Kingdom)	Reducing health inequalities is a core principle that guides development of NICE guidance and standards	Adapt simplified Labonte model to map factors that cause health inequalities, where they come from, and how they interact with each other as NICE provides guidance on interventions to focus on treating place as well as people	National Health Services Core 20 Plus 5 program recommendations for addressing five key clinical areas of health care inequalities in children, young people, and adults (with special attention to most deprived 20% of national population plus specifically chosen groups experiencing poorer-than-average health access/outcomes) Setting a lead role for integrated care systems in tackling health inequalities, focusing preventive services on certain ethnic groups experiencing poorer health Providing guidance, case studies, and further resources to help integrated care systems develop approaches to addressing health inequalities	^62–65^
Healthy People 2030 (U.S. Department of Health and Human Services)	Overarching goals include eliminating health disparities, achieving health equity, and attaining health literacy to improve the health and well-being of all, in this decade	Through systems approaches, guide diverse, distinct disease prevention and health promotion efforts throughout the U.S. toward these common goals Prioritize examining structural and systematic inequities that contribute to avoidable health disparities and to evolving social conditions rather than focusing on disease outcomes attributed to individual behaviors	Short- and long-term actions that: o Attend to the root causes of health inequalities and disparities, and to groups that have faced major obstacles to health o Promote equal opportunities for all persons to seek the highest possible level of health and well-being o Distribute socioeconomic resources in a way that progressively reduces health inequalities and disparities and maintain health equity once inequalities are eliminated Policies and practices that reduce or eliminate the root causes of health inequities and disparities Strategies that address structural inequities involving the full spectrum of SDoH Website offering: o Hundreds of evidence-based resources on addressing SDoH o Tips and tools on how to use resources in health care workplaces	^66–69^
U.S. Health Resources and Services Administration Maternal and Child Health Bureau	In the *Blueprint for Change: Guiding Principles for a System of Services for Children and Youth With Special Health Care Needs and Their Families*: Elimination of structural and systemic causal barriers to health equity (e.g., discrimination, poverty, other social risk factors) Design and implementation of all sectors, systems, and programs that fund, deliver, and monitor services and supports such that they reduce health disparities and improve health outcomes for all those they serve	Dismantle and replace policies and laws that systemize oppression at all levels Ensure that children and youth at risk of special health care needs are identified and supported to optimize their health outcomes Ensure system accountability through performance and outcome measures	Coordinating policies, practices, and procedures across sectors to address the root causes of health disparities and mitigate the health effects of societal oppression Collecting and sharing data on SDoH Developing a diverse workforce trained in evidence-informed, equitable, and culturally responsive delivery of service and supports	^70^
Office of Minority Health and Health Equity (U.S. FDA)	Vision: creating a world where health equity is a reality for all	Promote and protect the health of diverse populations through research and communication of science that addresses health disparities	Offering educational fact sheets, brochures, and infographics on common health concerns in lay terms in more than 15 languages Facilitating reporting of adverse events or problems to the FDA, including in Spanish Supporting multicultural professional organizations and minority health research Advancing minority health and health equity-focused regulatory science research, internships, and fellowships in collaboration with academia, minority-service institutions, government agencies, and nonprofit organizations Providing guidance on related aspects of clinical trial research (e.g., the collection of race and ethnicity data) Raising awareness around FDA initiatives and campaigns that advance minority health and health equity	^71,72^
American Public Health Association (APHA)	Creating health equity is a guiding priority and core value	Optimize the conditions in which people are born, grow, live, work, learn, and age Work with other sectors to address the factors that influence health, including employment, housing, education, health care, public safety, and food access Name racism as a force in determining how these social determinants are distributed	Publishing comprehensive book, *Racism: Science and Tools for the Public Health Professional* Providing Public Health and Equity Resource Navigator (PHERN), a curated hub of over 1600 resources covering key and emerging public health topics, equity, and antiracism (with the Alliance for Disease Prevention) Offering educational resources and initiatives in areas that impact public health (e.g., climate change) highlighting interplay with health equity Offering numerous racial equity resources including: o Webinar series on efforts to address systems, policies, and practices designed to limit opportunities for people of color o Suite of policies and practices for local leaders that are being implemented across the country to promote racial healing and address social inequities	^24,73–76^
American Society of Hematology (ASH)	ASH is committed to building and nurturing a global hematology community and workforce inclusive of diverse perspectives, talents, and experiences	Combat inequities in hematology by supporting scientists and clinicians from underrepresented backgrounds and embracing diverse voices across the patient and health care communities	Funding multiple student and trainee awards and grants to minorities in the field (ASH Minority Recruitment Initiative) Supporting women hematologists (Women in Hematology Working Group) Offering toolkits (ASH Health Equity Collective) to: o Assist sponsors to incorporate DEI principles throughout the clinical trial life cycle o Empower ASH members to drive DEI change and dismantle racism in their daily roles	^77,78^

APHA, American Public Health Association; ASH, American Society of Hematology; DEI, diversity, equity, and inclusion; FDA, U.S. Food and Drug Administration; NICE, National Institute for Health and Care Excellence; PHERN, Public Health and Equity Resource Navigator; SDoH, social determinants of health.

## Methods

### Logic model

**FIG. 1.**
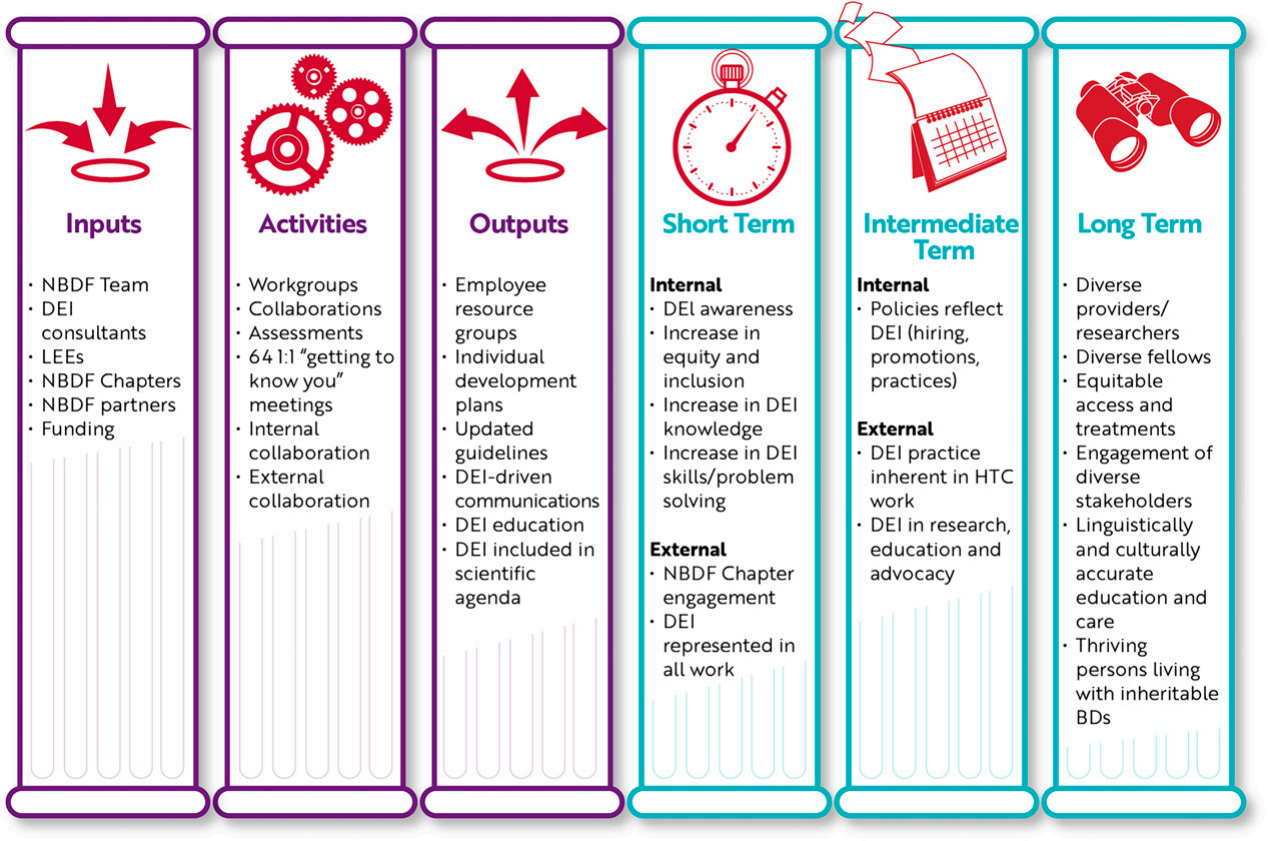
NBDF HEDI logic model. The left three pillars list inputs, activities, and outputs which NBDF anticipated incorporating into their HEDI work. The right three pillars list short-, intermediate-, and long-term outcomes logically expected to result from successful activation of the left three pillars. 64 1:1, sixty-four one-on-one meetings with health equity, diversity, and inclusion vice president; BD, blood and bleeding disorder; DEI, diversity, equity, and inclusion; HEDI, health equity, diversity, and inclusion; HTC, hemophilia treatment center; LEE, lived experience expert; NBDF, National Bleeding Disorders Foundation.

Development of the NBDF HEDI strategic direction began with the elucidation of a logic model establishing the short-, intermediate-, and long-term expectations of intended inputs, activities, and outputs (Fig. 1). The model was founded upon available data on health equity and disparities in the inheritable BD community (summarized in the Introduction), the lack thereof, and initial community consultations. To detail each pillar in the model, further extensive intentional community consultations were undertaken over 2 years.

### Community consultation

A Health Equity Summit convened diverse stakeholders (LEEs, caregivers, HCPs, NBDF and Chapter staff and leadership, industry, and not-for-profit organizations) in 2022 in Atlanta, GA. The Summit sought to hear the lived experiences of persons with inheritable BDs, understand health disparities and inequities affecting the community, reveal missing outcomes data, and identify interventions for each stakeholder group to advance health equity. Discussions concerning access, mental health, health system navigation, and payers/policy focused around four calls to action:

- Transform organizational culture and align daily work to achieve health equity.
- Address emerging inheritable BD community health needs, including issues with access to care, by supporting adaptable, innovative, outcome-focused, sustainable programs and services.
- Improve access to services and treatment by supporting integration and coordination of health services, HCPs, payers, and the public health sector.
- Expand stakeholder partnerships that lead to sustainable initiatives that eliminate health disparities.

Lack of input from young people (aged 18–24) prompted consultation with NBDF’s National Youth Leadership Institute^80^ via a focus group (2022). Informed interactions with small education groups (e.g., Black persons with inheritable BDs and social workers), individual Chapter initiatives, attending conferences, and in collaborative discussions with partner organizations (e.g., ATHN, National Organization for Rare Disorders, and pharmaceutical companies) complemented the Summit’s findings.

### SDoH analysis

**FIG. 2.**
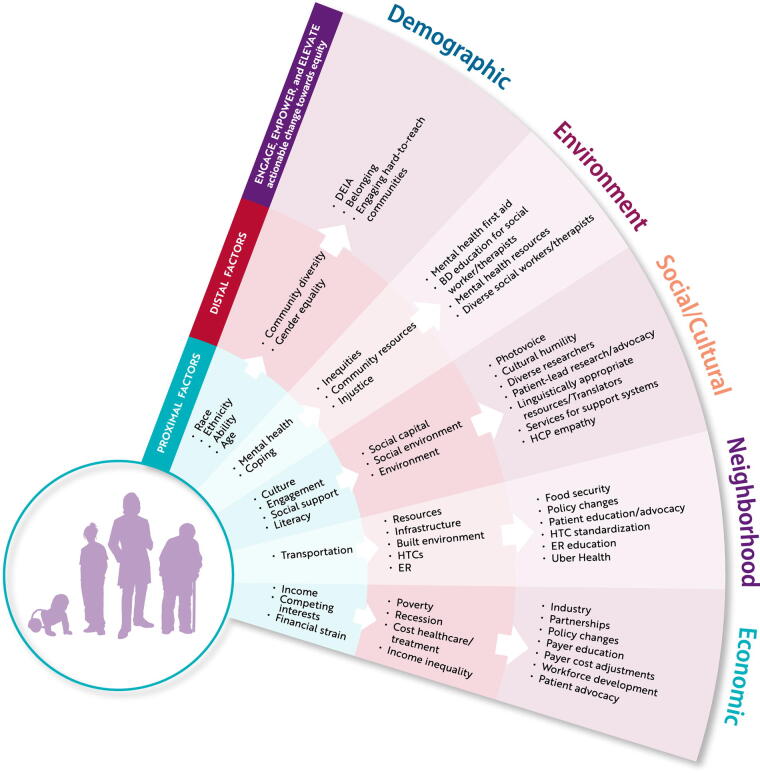
NBDF SDoH analysis. Analysis of community consultation input revealed proximal (inner arc) and distal (middle arc) SDoH factors that contribute to health disparities in the inheritable BD community throughout the lifespan and identified targets for actionable change to move the community toward health equity (outer arc) in demographic, environment, social/cultural, neighborhood, and economic domains. BD, blood and bleeding disorder; DEIA, diversity, equity, inclusion, and accessibility; ER, emergency room; HCP, health care provider; HTC, hemophilia treatment center; NBDF, National Bleeding Disorders Foundation; SDoH, social determinant of health.

The conceptual framework developed by Lund et al.^81^ to evaluate whether the United Nations Sustainable Development Goals address major social determinants of mental health was applied to the community data gathered concerning SDoH and health equity in the inheritable BD community (Fig. 2). This framework organizes SDoH into proximal factors: people, objects, or events in the immediate external environment with which an individual interacts, and distal factors: broader structural arrangements or societal trends, which exert an influence, often mediated by proximal factors,^82^ across four SDoH domains: demographic, environment, social/cultural, and economic.^81^ Proximal and distal SDoH factors that contribute to health disparities in the inheritable BD community throughout the lifespan were analyzed to identify targets for actionable change to engage, empower, and elevate the community toward health equity (Fig. 2).

A Health Equity Task Force with stakeholder representation as for the Summit was formed to:

- Discuss pressing issues around access and mental health, incorporating health system navigation and payer/policy elements.
- Produce viable solutions at individual, community, organizational, and institutional levels.
- Elevate the voice of LEEs in defining and offering solutions to further health equity.
- Shape the focus of future Health Equity Summits.

### E3 Model

In 2023, community roundtables with 25 Chapters spanning the United States further elaborated on the SDoH analysis, highlighting recurring themes and specific local contexts. The NBDF National Research Blueprint is an initiative to transform inheritable BD research^83,84^ in which K.L.N. chairs a HEDI working group. Insightful exchanges with LEE, Research and Development, Community Engagement, Workforce, Infrastructure, and Policy working groups detailed the importance and challenges of centering LEE expertise and HEDI principles throughout the research endeavor. The E3 Model: engaging, empowering, and elevating (Table 3) was developed to facilitate the co-creation of realizable action plans that can be tailored to capacity and provide easy access to accompanying resources and support. Constant community consultation continues to characterize the development of the NBDF’s HEDI strategy. Dialogue (e.g., with Chapters and staff, external partners, and HTCs) is central to the implementation of initiatives at all levels and provides essential feedback for the ongoing evolution of the strategy and its operationalization. Community roundtables, in English and Spanish, partnering closely with local Chapters and featuring the expertise of their LEEs, are instrumental in refining models and their application to individual communities. A second Health Equity Summit was held May 30–31, 2024, in New Orleans, LA. Discussing topics of access to care and the political determinants of health, delegates sought a better understanding of lived experiences, data outcomes, and evidence-based practices. The Summit’s findings have been published in a white paper.^85^

**Table 3.** The E3 Model: Engaging, Empowering, and Elevating (Dashboard Example)

SDoH*	Individual		Community	Organizational	Institutional
**Proximal**					
Race/Ethnicity		Embrace racial pride Practice self-advocacy Elevate own LEE voice	Practice antiracism Offer inclusion support groups Provide genetic counseling at HTCs	Conduct cultural humility trainings Develop inclusive health care settings Address inequities in diagnosis	Include women and minoritized populations in clinical trials Address racism in biomedical research Advance DEI efforts in medical programs
Engagement		Bring others to events with you to share the experience and provide support Design programs to put attendees at ease/increase their confidence in participating	Partner with other organizations Engage in community-based participatory research Increase rural outreach	Develop partnerships with HBCUs/HACUs Seek out work with nontraditional partners and bring them to the table	Facilitate LEE reporting of adverse events or problems Give a meaningful voice to LEEs on funding, regulatory, and policy decision-making bodies Commit to centering LEE voices across disease states/conditions
Literacy		Translate for a friend Offer free language classes	Offer translation services in more languages Provide closed captions on videos Do not assume understanding, rather meet people where they are and provide accessible information	Invest in ESL programs Offer interpreter apps on mobile devices Conduct more NBDF event sessions in Spanish	Offer infographics on common health concerns in non-English languages Establish guidelines and resources around creating materials in plain language Make graphic representations more accessible with integrated audio explanations
Transportation		Carpool with other community members	Provide gift cards for ride share programs (e.g., Uber/Lyft) Offer fuel (gas) gift cards Coordinate transportation through NBDF Chapters or a clinic travel program	Offer emergency financial assistance Fund and administer travel grants Deliver care from mobile health clinics	Create transportation support grants from HTC 340B^86^ funds
Income		Apply for financial assistance Investigate workforce development programs Access food assistance	Hold more free events Facilitate job training opportunities	Refer individuals to staffing agencies Connect persons to foundations offering financial assistance (e.g., PAN Foundation^87^)	Ensure staff are well versed in diverse organizations offering financial assistance and how to access them Host a job fair with local employers Expand WIOA state plans^88^
**Distal**					
Inequities		Practice self-advocacy Talk to local HTC social workers Engage in patient empowerment	Plan events to ensure they are accessible Conduct food drives Offer Spanish (or other non-English language) outreach programs	Utilize Healthy People 2030^67,69^ framework and resources Collaborate with intersectional organizations Apply the SDoH framework and initiatives^7^ to the organization	Improve care for incarcerated persons Conduct advocacy around chronic conditions Educate medical professionals on health inequities and contributing factors/practices
Built environment		Volunteer in a local organization Engage proactively with HTC/NBDF Chapter	Make specialized treatments (e.g., replacement clotting factor infusion) available in local hospitals Support farmer’s markets in rural areas Advocate for more, accessible public transit	Expand insurance network to cover more hospitals Prioritize convenience and access when choosing new hospital/clinic building sites Provide transportation benefits to employees Develop programs to eliminate neighborhood blight, enrich community resources	Advance interactive mapping app(s) for mobile devices Implement Complete Street^89^ policies Increase federal funding for public transit
Cost of health care and treatment		Discuss the cost of treatment and how it may restrict adherence with HCP (i.e., is recommended care affordable?) Access a patient assistance program Participate in CVR^90^	Attend state government advocacy days Share LEE stories Demonstrate how to use crowd funding platform (e.g., GoFundMe, CaringBridge)	Attend federal government advocacy days (e.g., NBDF Washington Days^91^) Develop resources to increase insurance health literacy (e.g., NBDF insurance toolkit^92^)	Institute universal health care Pass legislation on copay accumulators Expand Medicaid to broaden eligibility

* Examples of factors that might be highlighted by an SDoH analysis as in Figure 2 and selected by a specific community for their customized E3 Model action plan.

340B, 340B Drug Pricing Program (U.S. Health Resources and Services Administration); CVR, Community Voices in Research; E3, engaging, empowering, and elevating; ESL, English as a Second Language; HACUs, Hispanic Association of Colleges and Universities; HBCUs, Historically Black Colleges and Universities; HCP, health care provider; HTC, hemophilia treatment center; LEE, lived experience expert; NBDF, National Bleeding Disorders Foundation; PAN, Patient Access Network; WIOA, Workforce Innovation and Opportunity Act (U.S. Department of Labor).

## Results

### Key features of the NBDF’s HEDI strategic direction

#### Rooted in community

The NBDF’s HEDI strategic direction is rooted in community needs and expertise. LEEs constitute a key source of the knowledge foundation upon which the strategy is built; continuous learning from an ever-expanding diversity of LEEs is essential. Cultural humility and respectful curiosity are paramount in the relationship building and trust establishment prerequisite to receiving frank intelligence identifying true and pressing needs, and realizable solution opportunities. The guiding principle of meeting people where they are directs ongoing efforts to learn from and meet the needs of the full diversity of inheritable BD LEEs.

#### Vision, leadership, and guidance

The NBDF’s HEDI strategic direction provides vision, leadership, and guidance in addressing the complex multifaceted challenge of advancing health equity for the inheritable BD community. Applying a HEDI lens to all NBDF initiatives, policies, and communications sets a standard and expectations that Chapters, partners, and the broader community can look to. Recognizing the impossibility of advancing all worthwhile work simultaneously, NBDF analyzed the extensive intelligence gathered and identified priority populations and strategies to orient efforts. The 10-year strategic direction identifies five priority populations:

- Bilingual and multilingual populations
  - Over 240 million people speak English in the U.S.; however, many also speak Spanish (41.3 million), Chinese (3.4 million), Tagalog (1.72 million), Vietnamese (1.52 million), Arabic (1.39 million), French (1.18 million), Korean (1.07 million), Russian (1.04 million), and Portuguese (937,000).^93^
- Minoritized and marginalized populations
  - The NBDF Sate of the Science Working Group examining HEDI opportunities and challenges for the National Research Blueprint defined minoritized and marginalized populations to include “persons who have been traditionally underserved, excluded, and/or oppressed based on a given social standing or some characteristic including but not limited to race, ethnicity, sex, gender identity, sexuality, age, income, disability status, language, culture, faith, geographic location, and country of birth.”^39^
- Rural residents and those who are geographically isolated
  - More than 46 million Americans, or 15% of the population, live rurally and are thus at greater risk of, among other things, death from motor vehicle crashes and opioid overdoses, face greater community and family challenges for children with mental, behavioral, and development disorders, and could benefit from better access to health care services.^94^
- WGPPM
  - Individuals in this demographic with inheritable BDs face particular challenges receiving appropriate diagnosis and care and participating in research. Excessive uterine bleeding such as heavy menstrual bleeding and post-partum hemorrhage can impact the lives of anyone who has or had a uterus.^38^
- Young adults
  - A total of 21.6 million Americans identified as young adults in 2022.^95^ The transition from pediatric to adult care for people with inheritable BDs is laden with potential issues navigating the health care system, managing insurance coverage, and establishing new relationships with specialist HCPs (e.g., following relocation for work or study and seeking an adult rather than pediatric hematologist).^26^

Ten strategic approaches to dismantling the barriers to health equity in the inheritable BD community in 2024–2034 are proposed:

- Increasing access to care for all
- Translation of resources into the primary language of individuals with inheritable BDs
- Engaging unengaged populations
- Health and literacy, advancing plain language use
- Gender equity in testing and diagnosis, including improved diagnosis for WGPPM
- Improving access to mental health services and programs
- Practicing cultural humility with cultural competence
- SDoH: recognizing, integrating into care, and partnering to address
- Addressing systemic racism, discrimination, and unconscious bias
- Addressing policy needs for the inheritable BD community (e.g., insurance literacy and copay accumulators).

In establishing these priorities, NBDF defines a starting point, a long-term vision, and key milestones on the health equity journey for the inheritable BD community. The priorities encompass relatively straightforward initiatives that are easy to design, implement, and realize with rapid success, as well as more aspirational targets requiring decades of innovative work to achieve.

#### Customizable, realistic, relevant, supported

The principles of meeting people where they are and taking leadership from the expertise of those living with inheritable BDs also govern the operationalization of the HEDI strategic direction. Every individual, group, or community committed to doing the work of advancing health equity is expected to tailor their approach to their specific capacity and needs. The E3 Model offers a tool to detail customized concrete actions to be implemented at the individual, community, organizational, and institutional levels to address factors contributing to health disparities identified in an SDoH analysis (Fig. 2). NBDF puts the priorities outlined above at the community’s disposal and offers support, tools, and resources to facilitate the identification and implementation of initiatives they can realistically undertake and which address their most urgent needs. For example, they may work with a Chapter to analyze the proximal and distal SDoH factors operating locally to the detriment of health equity (Fig. 2) and develop a realistic action plan to engage, empower, and elevate health equity within their capacities (Table 3). NBDF can connect groups to the resources (e.g., translations of key documents developed by other Chapters and not-for-profit organizations specializing in specific SDoH such as homelessness or financial need), tools (e.g., the NBDF insurance toolkit^92^ and medical and scientific guidance documents^96^), and technical assistance they need to succeed. Much as one might consult an informatics expert to gain competence with an unfamiliar software package in their research or practice, so too can individuals or groups avail themselves of NBDF’s HEDI champions to accompany them through the acquisition and deployment of the new skills, knowledge, and perhaps mindset required to successfully undertake health equity work.

NBDF recognizes that efforts to advance health equity face limitations imposed by society and systems and must contend with the realities of policy, people, and politics. This approach of leadership, guidance, customization, resources, and support is designed to enable everyone at every level to work with a sense of investment, ownership, and pride with a shared commitment to the incorporation of diversity, equity, inclusion, and belonging values and strategies.

#### Constant evolution

The concrete actions identified in each customized E3 plan will be monitored for their impact, effectiveness, and shortcomings. Sharing learnings between groups addressing similar challenges or employing similar approaches will enrich the constant evolution of efforts. NBDF, as a central coordinating entity, can act as an intelligence conduit, maximizing the impact of learnings from each initiative, successful or otherwise. NBDF also looks forward to offering insights gleaned from collaborative efforts to advance health equity with partner organizations, nationally and internationally. Constant community consultation, at all levels, will continue to direct the NBDF’s HEDI strategy during and beyond 2024–2034.

## Conclusion

**FIG. 3.**
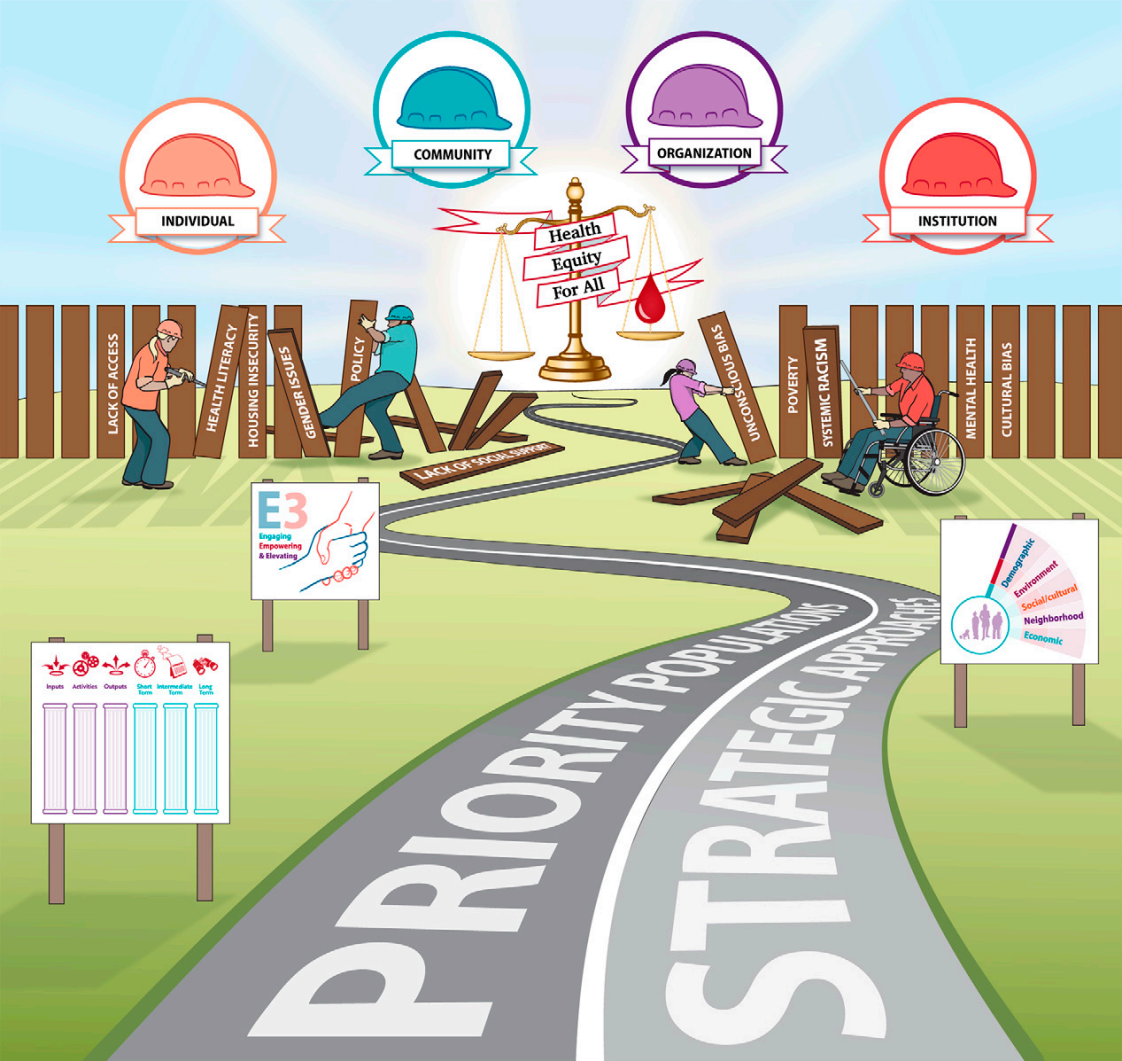
Identifying and dismantling barriers to social justice. NBDF developed a HEDI strategy that identified barriers to health equity in the inheritable BD community and targets actionable changes that engage, empower, and elevate the community through health equity to social justice. BD, blood and bleeding disorder; HEDI, health equity, diversity, and inclusion; NBDF, National Bleeding Disorders Foundation.

Analysis of how specific SDoH factors cause health disparities and inequities in a rare disorder community led to the identification of the necessary engagement, empowerment, and elevating work, and detailed strategic objectives and tactics, to advance toward justice (Fig. 3).

Drawing upon limited available data, extensive community consultation, and a thorough landscape scan, NBDF identified SDoH impacting the inheritable BD community, naming the planks of the fence impeding attainment of health equity. Application of the practical E3 Model details the engaging, empowering, and elevating work individual, community, organizational, and institutional stakeholders must undertake to eliminate each plank, thus dismantling the fence. The establishment of overarching priorities and strategies provides leadership, guidance, and vision, whereas offering support, tools, expertise, and resources facilitates tailoring to each community’s capacity and reality.

Other rare disorder groups can implement this approach to develop their own HEDI strategic direction and advance health equity and social justice for their respective communities.

## Abbreviations Used

340B: 340B Drug Pricing Program (U.S. Health Resources & Services Administration)
APHA: American Public Health Association
ASH: American Society of Hematology
ATHN: American Thrombosis and Hemostasis Network
BD: Bleeding Disorder
CVR: Community Voices in Research
DEI: Diversity, Equity, and Inclusion
DEIA: Diversity, Equity, Inclusion, and Accessibility
E3: Engaging, Empowering, and Elevating
ER: Emergency Room
ESL: English as a Second Language
FDA: U.S. Food and Drug Administration
HACU: Hispanic Association of Colleges and Universities
HBCU: Historically Black Colleges and Universities
HCP: Health Care Provider
HEDI: Health Equity, Diversity, and Inclusion
HTC: Hemophilia Treatment Center
LEE: Lived Experience Expert
NBDF: National Bleeding Disorders Foundation
NICE: National Institute for Health and Care Excellence
PAN: Patient Access Network
PHERN: Public Health & Equity Resource Navigator
SDoH: Social Determinant(s) of Health
U.S.: United States
VWD: Von Willebrand Disease
WGPPM: Women, Girls, and People who have or had the Potential to Menstruate
WHO: World Health Organization
WIOA: Workforce Innovation and Opportunity Act (U.S. Department of Labor)
